# Supplementary material for: Bidirectional Associations between Parental Non-Responsive Feeding Practices and Child Eating Behaviors: A Systematic Review and Meta-Analysis of Longitudinal Prospective Studies
Source: Nutrients. 2022 Apr 30;14(9):1896. doi: 10.3390/nu14091896 (PMC9103127; doi:10.3390/nu14091896)
Supplement: Supplementary file 1 [file nutrients-14-01896-s001.zip › nutrients-1685810-supplementary.pdf]

Table S1. Quality appraisal by the JBI Critical Appraisal Checklist for Cohort Studies

First author, year	Q1	Q2	Q3	Q4	Q5	Q6	Q7	Q8	Q9	Q10	Q11	Overall quality rating
Jansen et al 2018 [14]	NA	NA	Y	Y	Y	NA	Y	Y	N	Y	Y	P
Jansen et al 2017 [31]	NA	NA	Y	Y	Y	NA	Y	Y	N	Y	Y	P
Lumeng et al 2018 [34]	NA	NA	Y	N	N	NA	Y	Y	N	Y	Y	M
Mallan et al 2018 [66]	NA	NA	Y	Y	Y	NA	Y	Y	N	Y	Y	P
Steinsbekk et al 2016 [32]	NA	NA	Y	Y	Y	NA	Y	Y	N	Y	Y	P
Rodgers et al 2013 [30]	NA	NA	Y	N	N	NA	Y	Y	N	N	Y	M
Bergmeier et al 2015 [40]	NA	NA	Y	Y	Y	NA	Y	Y	Y	N	Y	P
Gregory et al 2010 [52]	NA	NA	UN	Y	Y	NA	Y	Y	N	Y	Y	P
Bjørklund et al 2018 [64]	NA	NA	Y	Y	Y	NA	Y	Y	Y	Y	Y	P
Bergmeier et al 2014 [67]	NA	NA	Y	Y	Y	NA	Y	Y	Y	Y	Y	P
Zohar et al 2020 [62]	NA	NA	Y	Y	Y	NA	Y	Y	Y	Y	Y	P
Jansen et al 2020 [53]	NA	NA	Y	Y	Y	NA	Y	Y	N	N	Y	M
Berge et al 2020 [63]	NA	NA	Y	Y	Y	NA	Y	Y	Y	Y	Y	P
Costa et al 2021[65]	NA	NA	Y	Y	Y	NA	Y	Y	Y	Y	Y	P

Notes. N, negative study quality NA, not applicable; P, positive study quality; M, median study quality; UN, unclear; Y, yes.

Q1. Were the two groups similar and recruited from the same population?

Q2. Were the exposures measured similarly to assign people to both exposed and unexposed groups?

Q3. Was the exposure measured in a valid and reliable way?

Q4. Were confounding factors identified?

Q5. Were strategies to deal with confounding factors stated?

Q6. Were the groups/participants free of the outcome at the start of the study (or at the moment of exposure)?

Q7. Were the outcomes measured in a valid and reliable way?

Q8. Was the follow up time reported and sufficient to be long enough for outcomes to occur?

Q9. Was follow up complete, and if not, were the reasons to loss to follow up described and explored?

Q10.Were strategies to address incomplete follow up utilized?

Q11.Was appropriate statistical analysis used?

Table S2. Longitudinal effects from parental non-responsive feeding practices to child eating behaviors (F→E)

	Food fussiness (FF)	Food responsiveness	Satiety responsiveness	Emotional eating	Enjoyment of food
**Restriction**	ϕ(2 y-3.7 y) [66] β= 0.06<br>ϕ(3.7 y-5 y) [66] β= -0.01<br>ϕ(3.3 y-4.3 y) [52] β=0.04<br>ϕ(3.09 y-4.09 y) [40] β= NR<br>ϕ(3 y-4 y) [67] β= 0.03<br>ϕ(3.3y-4.3y)[63] β=-0.07, 95% CI (-0.14, 0.001)<br>ϕ(4.3y-5.3y)[63] β=-0.03, 95% CI (-0.10, 0.04)<br>ϕ(5.3y-6.3y)[63] β=-0.01, 95%CI (-0.08, 0.06)<br>ϕ(4.3y-5.3y)[62] β=-0.007, t=-0.07, *P*=0.94<br>ϕ(4 y- 7 y)[65] β=0.013, 95% CI (-0.017, 0.043)	ϕ(2 y- 3.7 y) [14] β=NR<br>ϕ(3.7 y- 5 y) [14] β= NR<br>ϕ(6 y-8 y) [32] β=0.04, 95% CI (-0.05, 0.13)<br>ϕ (3.3 y-4.3 y) [52] β=0.06<br>ϕ(6 y-8 y) [64] β=0.03, 95% CI (-0.07, 0.12)<br>ϕ(3.3y-4.3y)[63] β=0.04, 95% CI (-0.04, 0.12)<br>ϕ(4.3y-5.3y)[63] β=0.03, 95% CI (-0.05, 0.11)<br>ϕ(5.3y-6.3y)[63] β=0.04, 95%CI (-0.03, 0.12)<br>ϕ(4 y- 7 y)[65] β=-0.011, 95% CI (-0.019, 0.041)	ϕ (2 y- 3.7 y) [14] β= NR<br>ϕ (3.7 y- 5 y) [14] β= NR<br>ϕ(6 y-8 y) [32] β=0.05, 95% CI (-0.07, 0.16)<br>ϕ(3.3y-4.3y)[63] β=-0.05, 95% CI (-0.13, 0.02)<br>ϕ(4.3y-5.3y)[63] β=-0.02, 95% CI (-0.09, 0.06)<br>ϕ(5.3y-6.3y)[63] β=-0.04, 95%CI (-0.12, 0.04)	ϕ(6 y-8 y) [32] β=-0.04, 95% CI (-0.14, 0.07)	ϕ(6 y-8 y) [32] β=0.05, 95% CI (-0.06, 0.16)<br>ϕ(3 y-4 y) [67] β=0.02<br>ϕ(3.3 y-4.3 y) [52] β= -0.02<br>-(2.9 y-3.9 y) [40] β=-0.09, SE:0.04<br>ϕ(3.3y-4.3y)[63] β=0.06, 95% CI (-0.01, 0.14)<br>ϕ(4.3y-5.3y)[63] β=0.03, 95% CI (-0.05, 0.11)<br>ϕ(5.3y-6.3y)[63] β=0.04, 95%CI (-0.03, 0.12)
**Pressure to eat**	＋(4 y- 6 y) [31] β=0.17, 95% CI (0.14, 0.20)<br>ϕ(21 m-27 m) [34] β=0.089 (FF Measure: CEBQ)<br>ϕ(27 m-33 m) [34] β=0.094 (FF Measure: CEBQ)<br>ϕ(21 m-27 m) [34] β=0.040 (FF Measure: BAMBI)<br>ϕ(27 m-33 m) [34] β=0.089 (FF Measure: BAMBI)<br>ϕ (3.3 y-4.3 y) [52] β=0.01<br>ϕ(3.09 y-4.09 y) [40] β= NR<br>ϕ(3 y-4 y) [67] β=0.11<br>ϕ(4.3y-5.3y)[62] β=0.08, t=0.86, *P*=0.39<br>+(4 y- 7 y)[65] β=0.033, 95% CI (0.022, 0.064)	ϕ(3.3 y-4.3 y) [52] β=0.00<br>+(4 y- 7 y)[65] β=-0.096, 95% CI (-0.126, -0.065)			ϕ(3 y-4 y) [67] β=0.04<br>-3(3.3 y-4.3 y) [52] β= -0.25<br>-(2.9 y-3.9 y) [40] β=-0.09, SE:0.07

		Food fussiness (FF)	Food responsiveness	Satiety responsiveness	Emotional eating	Enjoyment of food
**Use food as a reward**	**Reward (behavior)**	ϕ(2 y-3.7 y) [66] β= 0.02<br>+(3.7 y- 5 y) [66] β= 0.08	+(2 y-3.7 y) [14] β= 0.12*, P=0.025<br>ϕ(3.7 y- 5 y) [14] β=NR	ϕ(2 y- 3.7 y) [14] β=NR<br>ϕ(3.7 y- 5 y) [14] β=NR		
	**Reward (eating)**	ϕ(2 y-3.7 y) [66] β= -0.06<br>ϕ(3.7 y- 5 y) [66] β= -0.02	ϕ(2 y- 3.7 y) [14] β=NR<br>ϕ (3.7 y- 5 y) [14] β=NR	ϕ(2 y- 3.7 y) [14] β=NR<br>ϕ(3.7 y- 5 y) [14] β=NR		
**Use food as a reward**		+(4-9 y)[53] β=0.06, 95% CI (0.03, 0.09)<br>ϕ(3.3y-4.3y)[63] β=0.05, 95% CI (-0.02, 0.12)<br>ϕ(4.3y-5.3y)[63] β=-0.02, 95% CI (-0.09, 0.05)<br>ϕ(5.3y-6.3y)[63] β=-0.01, 95%CI (-0.08, 0.06)	+(6 y-8 y) [32] β= 0.11, 95% CI (0.02, 0.20)<br>ϕ(6 y-8 y) [64] β= -0.02, 95% CI (-0.12, 0.07)<br>ϕ(4-9 y)[53] β=-0.02, 95% CI (-0.05, 0.016)<br>ϕ(3.3y-4.3y)[63] β=0.06, 95% CI (-0.02, 0.14)<br>+(4.3y-5.3y)[63] β=0.10, 95% CI (0.01, 0.18)<br>+ (5.3y-6.3y)[63] β=0.10, 95% CI (0.02, 0.18)	ϕ(6 y-8 y) [32] β= -0.01, 95% CI (-0.09,0.08)<br>ϕ(4-9 y)[53] β=0.02 95% CI (-0.01, 0.05)<br>ϕ(3.3y-4.3y)[63] β=0.07, 95% CI (-0.01, 0.14)<br>ϕ(4.3y-5.3y)[63] β=0.01, 95% CI (-0.07, 0.09)<br>ϕ(5.3y-6.3y)[63] β=0.02, 95%CI (-0.05, 0.10)	+(6 y-8 y) [32] β= 0.13, 95% CI (0.06, 0.20)<br>+(4-9 y)[53] β=0.07, 95% CI (0.04, 0.11)	ϕ(6 y-8 y) [32] β=0.06, 95% CI (-0.03, 0.14)<br>ϕ(3.3y-4.3y)[63] β=-0.05, 95% CI (-0.13, 0.02)<br>ϕ(4.3y-5.3y)[63] β=0.03, 95% CI (-0.05, 0.11)<br>ϕ(5.3y-6.3y)[63] β=0.001, 95%CI (-0.07, 0.08)
**Emotional feeding**		ϕ(3.3y-4.3y)[63] β=0.04, 95% CI (-0.04, 0.11)<br>ϕ(4.3y-5.3y)[63] β=-0.06, 95% CI (-0.13, 0.01)<br>ϕ(5.3y-6.3y)[63] β=-0.01, 95%CI (-0.08, 0.06)	ϕ(3.3y-4.3y)[63] β=0.03, 95% CI (-0.05, 0.12)<br>+(4.3y-5.3y)[63] β=0.10, 95% CI (0.02, 0.18)<br>+(5.3y-6.3y)[63] β=0.09, 95%CI (0.01, 0.17)	+(3.3y-4.3y)[63] β=0.08, 95% CI (0.005, 0.16)<br>-(4.3y-5.3y)[63] β=-0.09, 95% CI (-0.17, -0.005)<br>ϕ(5.3y-6.3y)[63] β=0.08, 95% CI (-0.004, 0.16)	+(1.5 y-2.5 y) [30] β=0.14, *P* = 0.012	ϕ(3.3y-4.3y)[63] β=-0.08, 95% CI (-0.16, 0.004)<br>ϕ(4.3y-5.3y)[63] β=0.08, 95% CI (-0.01, 0.16)<br>ϕ(5.3y-6.3y)[63] β=0.02, 95%CI (-0.06, 0.10)

Notes. FF: food fussiness. +positive association, -negative association, ϕnon-significant association, NR not reported data

Table S3. Longitudinal effects from child eating behaviors to parental non-responsive feeding practices (E→F)

	Restriction	Pressure to eat	Use food as a reward		Use food as a reward	Emotional feeding
			Reward for eating	Reward for behavior		
**Food fussiness**	ϕ(2 y-3.7 y) [66] β=0.04<br>ϕ(3.7 y-5 y) [66] β=0.11<br>ϕ(3.3y-4.3y)[63] β=-0.01, 95% CI (-0.09, 0.06)<br>ϕ(4.3y-5.3y)[63] β=0.01, 95% CI (-0.07, 0.08)<br>ϕ(5.3y-6.3y)[63] β=0.004, 95%CI (-0.07, 0.08)<br>ϕ(4 y- 7 y)[65] β=-0.009, 95% CI (-0.035, 0.018)	+ (1.5 y-4 y) [31] β=0.18, 95% CI (0.15, 0.21)<br>+ (3 y-4 y) [31] β=0.24, 95% CI (0.21, 0.26)<br>ϕ(21m-27m) [34]β=0.030 (Measure: CEBQ)<br>ϕ(27m-33m) [34]β=0.055 (Measure: CEBQ)<br>ϕ(21m-27m) [34]β=0.003 (Measure: BAMBI)<br>ϕ(27m-33m) [34]β=-0.016 (Measure: BAMBI)<br>+(4 y- 7 y)[65]β=0.060, 95% CI (0.034, 0.086)	ϕ(2 y-3.7 y) [66] β=0.09<br>+ (3.7 y-5 y) [66] β=0.13	ϕ(2 y-3.7 y) [66] β=-0.06<br>ϕ(3.7 y-5 y) [66] β=0.04	ϕ(4-9 y)[53] β=0.03, 95% CI (-0.01, 0.06)<br>ϕ(3.3y-4.3y)[63] β=-0.01, 95% CI (-0.09, 0.07)<br>ϕ(4.3y-5.3y)[63] β=0.06, 95% CI (-0.01, 0.14)<br>ϕ(5.3y-6.3y)[63] β=-0.02, 95%CI (-0.09, 0.05)	ϕ(3.3y-4.3y)[63] β=0.02, 95% CI (-0.04, 0.09)<br>ϕ(4.3y-5.3y)[63] β=-0.05, 95% CI (-0.11, 0.02)<br>-(5.3y-6.3y)[63] β=-0.06, 95%CI (-0.12, -0.01)
**Food responsiveness**	ϕ(2 y-3.7 y) [14] β=NR<br>ϕ(3.7 y-5 y) [14] β=NR<br>ϕ(6 y-8 y) [32] β=0.02, 95% CI (-0.06, 0.09)<br>ϕ(3.3y-4.3y)[63] β=0.04, 95% CI (-0.04, 0.11)<br>ϕ(4.3y-5.3y)[63] β=0.04, 95% CI (-0.03, 0.12)<br>ϕ(5.3y-6.3y)[63] β=0.03, 95%CI (-0.05, 0.10)<br>+(4 y- 7 y)[65]β=0.047, 95% CI (0.019, 0.075)	-(4 y- 7 y)[65] β=-0.044, 95% CI (-0.071, -0.016)	ϕ(2 y-3.7 y) [14] β=NR<br>ϕ(3.7 y-5 y) [14] β=NR	ϕ(2 y-3.7 y) [14] β=NR<br>ϕ(3.7 y-5 y) [14] β=NR	ϕ (6 y-8 y) [32] β=0.07, 95% CI (-0.03, 0.17)<br>+(4-9 y)[53] β=0.04, 95% CI (0.01, 0.08)<br>+(3.3y-4.3y)[63] β=0.10, 95% CI (0.03, 0.18)<br>ϕ (4.3y-5.3y)[63] β=0.06, 95% CI (-0.02, 0.14)<br>+ (5.3y-6.3y)[63] β=0.10, 95% CI (0.03, 0.18)	ϕ(3.3y-4.3y)[63] β=0.04, 95% CI (-0.03, 0.10)<br>ϕ(4.3y-5.3y)[63] β=0.05, 95% CI (-0.02, 0.11)<br>ϕ(5.3y-6.3y)[63] β=0.05, 95%CI (-0.01, 0.10)
**Satiety responsiveness**	ϕ(2 y-3.7 y) [14] β=NR<br>+(3.7 y-5 y) [14] β=0.14, *P* = 0.010<br>ϕ(6 y-8 y) [32] β=-0.07, 95% CI (-0.16, 0.01)		ϕ(2 y-3.7 y) [14] β=NR<br>ϕ(3.7 y-5 y) [14] β=NR	ϕ(2 y-3.7 y) [14] β=NR<br>ϕ(3.7 y-5 y) [14] β=NR	ϕ(6 y-8 y) [32] β=0.05, 95% CI (-0.04, 0.13)<br>ϕ(4-9 y)[53] β=-0.01, 95% CI (-0.04, 0.02)<br>-(3.3y-4.3y)[63] β=-0.08, 95%	ϕ(3.3y-4.3y)[63] β=0.03, 95% CI (-0.04, 0.09)<br>ϕ(4.3y-5.3y)[63] β=-0.05, 95% CI (-0.12, 0.01)

	Restriction	Pressure to eat	Use food as a reward		Use food as a reward	Emotional feeding
			Reward for eating	Reward for behavior		
	ϕ(3.3y-4.3y)[63] β=-0.06, 95% CI (-0.14, 0.01)<br>ϕ(4.3y-5.3y)[63] β=-0.01, 95% CI (-0.08, 0.06)<br>ϕ(5.3y-6.3y)[63] β=-0.05, 95%CI (-0.13, 0.02)				CI (-0.15, -0.0004)<br>ϕ(4.3y-5.3y)[63] β=0.04, 95% CI (-0.04, 0.11)<br>ϕ(5.3y-6.3y)[63] β=0.01, 95%CI (-0.07, 0.08)	ϕ(5.3y-6.3y)[63] β=0.01, 95% CI (-0.05, 0.07)
**Enjoyment of food**	ϕ(6 y-8 y) [32] β=-0.02, 95% CI (-0.09, 0.05)<br>ϕ(3.3y-4.3y)[63] β=0.05, 95% CI (-0.02, 0.13)<br>ϕ(4.3y-5.3y)[63] β=-0.01, 95% CI (-0.08, 0.07)<br>ϕ(5.3y-6.3y)[63] β=0.04, 95%CI (-0.03, 0.12)				ϕ(6 y-8 y) [32] β=0.02, 95% CI (-0.06, 0.11)<br>ϕ(3.3y-4.3y)[63] β=0.04, 95% CI (-0.04, 0.12)<br>ϕ(4.3y-5.3y)[63] β=-0.04, 95% CI (-0.12, 0.03)<br>+(5.3y-6.3y)[63] β=0.08, 95%CI (0.01, 0.15)	ϕ(3.3y-4.3y)[63] β=-0.003, 95% CI (-0.07, 0.06)<br>ϕ(4.3y-5.3y)[63] β=0.06, 95% CI (-0.001, 0.13)<br>ϕ(5.3y-6.3y)[63] β=0.03, 95%CI (-0.02, 0.09)
**Emotional eating**	ϕ(6 y-8 y) [32] β=-0.08, 95% CI (-0.16, 0.00)				ϕ(6 y-8 y) [32] β=-0.02, 95% CI (-0.13, 0.09)<br>+(4-9 y)[53] β=0.06, 95% CI (0.03, 0.09)	+(1.5 y-2.5 y) [30] β=0.13, *P*=0.047

Notes. +positive association, -negative association, ϕnon-significant association, NR not reported data.
